# Greasy Cations Bind to Neutral Macromolecules in Aqueous Solution

**DOI:** 10.1021/acs.jpclett.4c00925

**Published:** 2024-06-05

**Authors:** Umay Eren Ertekin, Halil Ibrahim Okur

**Affiliations:** † Department of Chemistry, Faculty of Science, Bilkent University, 06800 Ankara, Turkey; ‡ National Nanotechnology Research Center (UNAM), Bilkent University, 06800 Ankara, Turkey

## Abstract

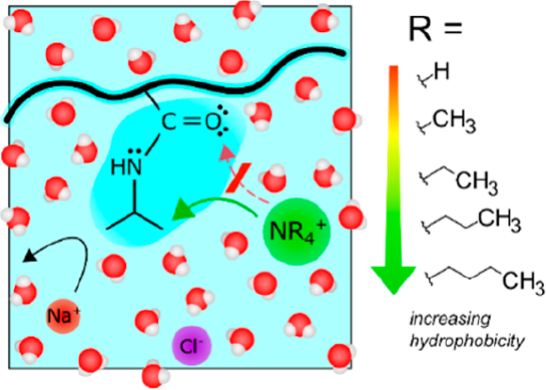

Ions influence the solution properties of macromolecules. Although much is known about anions, cationic effects are considered mostly in terms of weak interactions or exclusion from neutral interfaces. Herein, we have systematically studied the effect of quaternary tetraalkylammonium cations (NH_4_^+^, NMe_4_^+^, NEt_4_^+^, NPr_4_^+^, NBu_4_^+^) on the phase transition of poly(*N*-isopropylacrylamide) (PNIPAM) in aqueous solution. Solubility measurements were coupled to ^1^H NMR and ATR-FTIR spectroscopic measurements. The solubility and NMR measurements revealed a direct binding between the greasiest cations and the isopropyl group of the macromolecule, evidenced from the nonlinear, Langmuir-type chemical shift response only at the isopropyl NMR signals with increasing salt concentrations. The ATR-FTIR measurements focusing on the amide oxygen showed that it is not the main direct-binding site. Additionally, the salting-out effects of the greasier cations correlate with their hydration entropies. These results demonstrate that the most weakly hydrated cations can bind to macromolecules as strongly as the weakly hydrated Hofmeister anions.

It has been over a century since Franz Hofmeister demonstrated the specific effects of various salts on the solubility of proteins.^1,2^ The same apparent ionic series in the effects on protein solubility has since been observed in contexts as diverse as macromolecular stability, self-organization, interactions with surfaces, micellization, bubble coalescence, foam stability, and even thundercloud electrification.^3−14^ More recently, the mechanisms of specific ion effects have been investigated, wherein anions have often been ascribed the more dominant role. The general ordering of ions according to salting-out strength, a typical “direct” Hofmeister series for strong to weakly hydrated anions and for cations, can be given as^15,16^
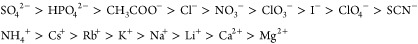


With advancements in spectroscopic, thermodynamic, and computational methods, a resurgence in the study of specific ion effects has produced new molecular-level models of Hofmeister effects mechanisms.^15^ On the anionic side, salting-out ions have been correlated with concentration induced surface tension elevation, and an overall ion depletion from macromolecular surfaces has been implicated as a key driving force.^16,17^ Weakly hydrated anions that effect salting-in have a lesser depletion from interfaces and exhibit affinity for binding interactions at specific sites on neutral macromolecules.^18,19^ Larger weakly hydrated anions, such as those typically used for ionic liquids have been shown to cause strong salting-in effects.^20^ Recent work has continued to expand upon the diversity of specific ion effects, as exemplified by developments such as the proposed “chaotrope effect”,^20^ characteristic in strong binding to hydrophobic hosts and pockets,^21−24^ and the surprising behavior of “superchaotropic” nanoions, which exhibit high affinity for neutral surfaces, cell membranes, and macrocyclic hosts.^21,25−29^

In contrast to the anionic effects, the chemistry of the Hofmeister cations has frequently been understated, often classified as passive counterions or presented as interacting only electrostatically with charged groups on proteins.^15,30−32^ Some metal cations (e.g., K^+^, Cs^+^) are indeed experimentally observed to act mainly as counterions that contribute a moderately strong salting-out effect onto neutral macromolecules.^33^ However, strongly hydrated cations such as Mg^2+^ or Ca^2+^ deviate from this trend: spectroscopic data, supported by computational studies, points to their weak interaction with amide oxygens.^34,35^ With neutral macromolecules, the same strongly hydrated cations manifest this propensity for weak yet observable specific interaction in partial salting-in contributions that become sizable at high salt concentrations.^33^ This is supported by simulation, which has indicated that solvent-shared cation–anion pairs mediate the local accumulation of the strongly hydrated cations around amide oxygens.^33^

The role of cations, however, is more complex and includes, for example, the recently demonstrated ion-specific tendency for ion pair formation in the bulk solution and the resulting modulation of the dominant anionic effects.^36,37^ Moreover, the counteranion-dependent stabilization and destabilization effects of guanidinium cation have also been demonstrated.^38,39^ The weakly hydrated class of cations in particular has not been given due inquiry. The typical cation ordering is limited solely to a handful of common, noninteracting ions. In contrast to anions, general interactions with neutral polar or hydrophobic macromolecular moieties in solution remain experimentally unprecedented for cations, beyond sparse hints of the salting-in action of some salts in the literature, generally attributed to their surfactant-like properties.^3,40,41^ To this end, we present herein a systematic study of a series of quaternary tetraalkylammonium salts. In incrementally varying the alkyl chain length of the symmetrical tetraalkylammonium cations and keeping a constant chloride counterion, the cationic effects were elucidated across the largely unexplored, more weakly hydrated side of the cationic Hofmeister series.

We conducted phase transition measurements of the thermoresponsive poly(*N*-isopropylacrylamide) (PNIPAM) as a function of tetraalkylammonium chloride salts and observed concentration-dependent elevation of lower critical solution temperatures (LCSTs) indicative of salting-in behavior, with magnitudes comparable to those of strongly binding weakly hydrated classical Hofmeister anions, e.g., SCN^–^ and I^–^, and a correlation with the cation hydrophobicity. Subsequently, ATR-FTIR spectroscopy was conducted for the soluble macromolecule below the LCST to examine interactions at the amide oxygen as a potential binding site. To elucidate the nature of the interaction, quantitative ^1^H NMR spectroscopy was employed, whereby NMR titrations that scan all sites on the macromolecule revealed that the terminal methyl of the pendant isopropyl groups were the main binding sites. As depicted in Figure 1, these larger, more weakly hydrated cations indeed diverge from the previously known strongly hydrated cationic mechanism for binding to neutral macromolecules and operate via different interactions at low to intermediate concentrations.

**Figure 1:**
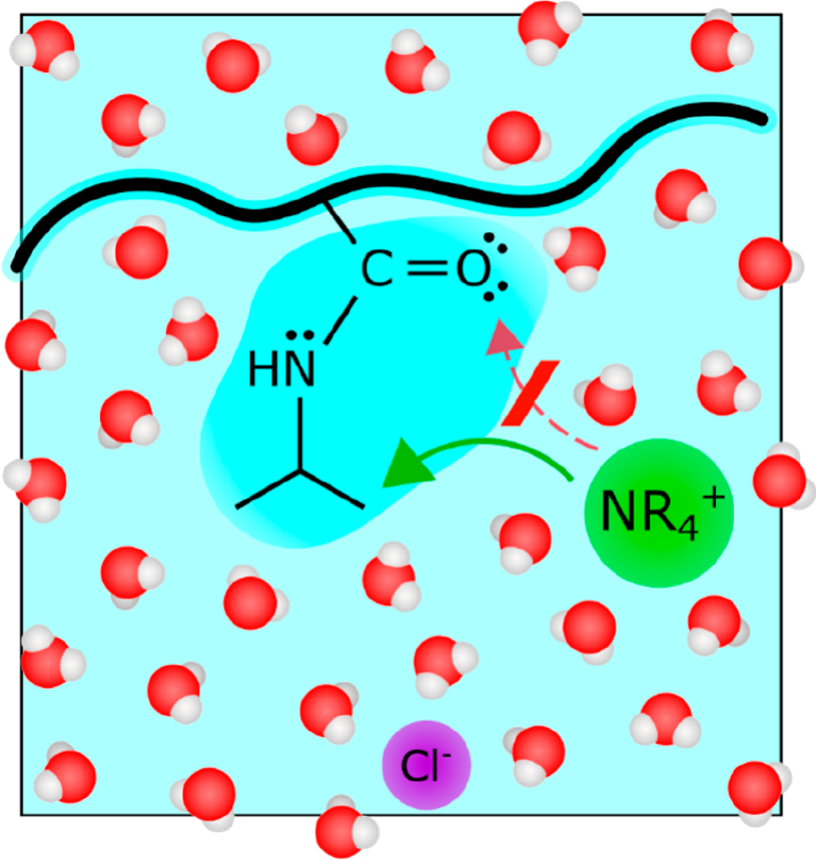
Schematic view of the route of interaction between greasy cations (NR_4_^+^ where R = Hydrogen, Methyl, Ethyl, *n*-Propyl, *n*-Butyl) and the polymer poly(*N*-isopropylacrylamide) (PNIPAM). Owing to their dual cationic-hydrophobic nature, these ions may preferentially interact with the carbonyl oxygen or the hydrophobic isopropyl groups.

Figure 2 shows the LCST plot of PNIPAM with increasing concentrations of NaCl, NaSCN, and five NR_4_Cl salts (where R = H, Me, Et, *n*-Pr, *n*-Bu) in aqueous solution. In the absence of salt, the LCST of PNIPAM is 33.05 °C. NaCl and NH_4_Cl alter the LCST almost linearly, with a slight curvature only at elevated salt concentrations. Note that this slight upward curvature was recently explored for various metal chloride salts.^33^ The smallest tetraalkylammonium salt, NMe_4_Cl, exhibits a similar, linear trend with a slope marginally shallower than NH_4_Cl, and thus, NMe_4_Cl also predominantly salts-out the macromolecule. Starting with NEt_4_Cl, however, the LCST profile changes remarkably: a significant deviation from linearity appears at low (0–0.4 M) salt concentrations. The apparent NPr_4_Cl salt effect on the LCST of PNIPAM is even stronger, with sharper nonlinearity. The salt of the greasiest cation, NBu_4_Cl, influences the LCST trend similarly, where the nonlinearity is significant enough that an LCST maximum is attained at 0.2 M, and a steeper downward curvature is also observed.

**Figure 2:**
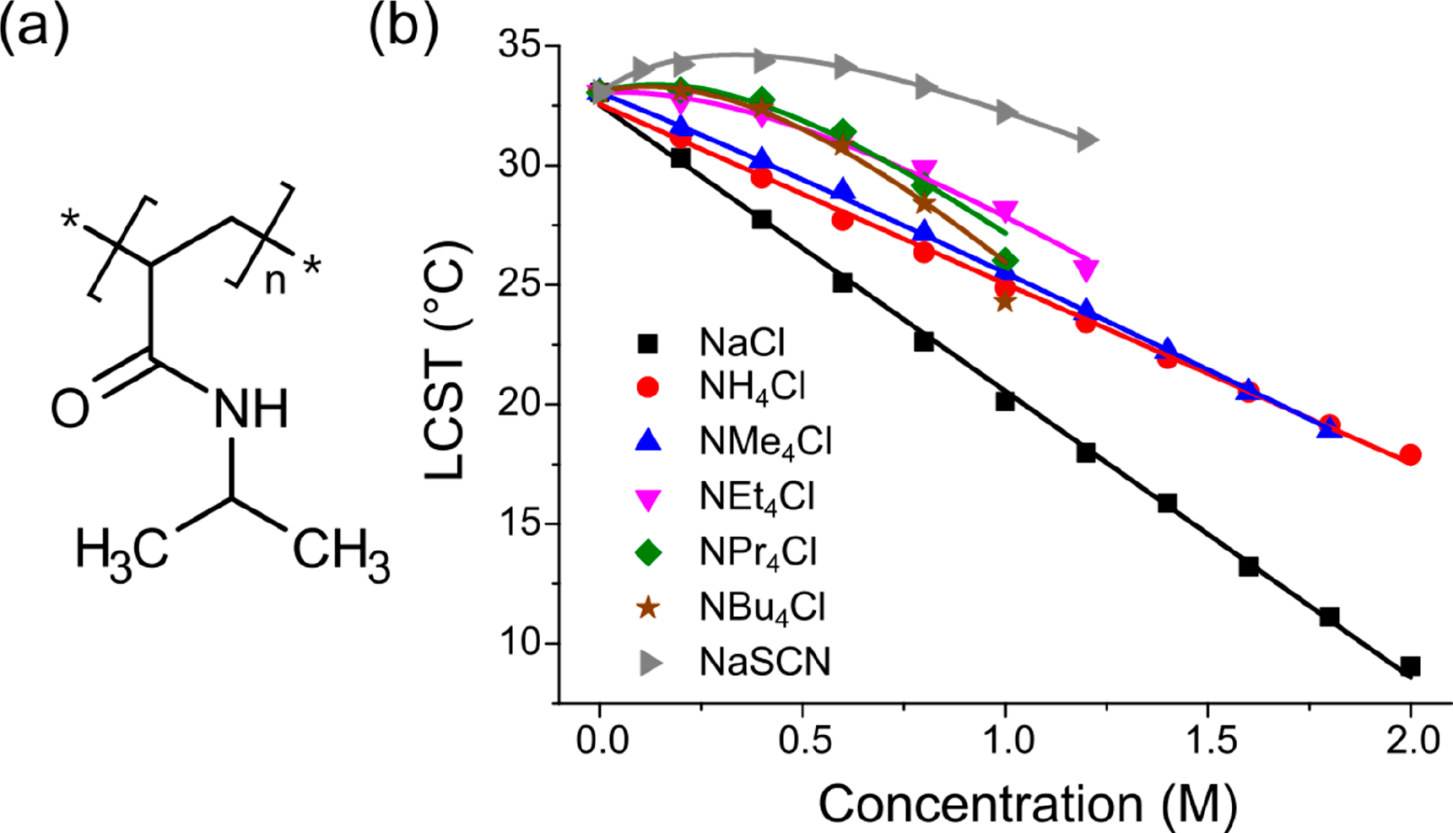
(a) Structure of poly(*N*-isopropylacrylamide) (PNIPAM). (b) Fitted lower critical solution temperature (LCST) curves for 5 mg/mL PNIPAM as a function of the salt concentration. Employed salts are given in the legend. The standard deviations of at least three measurements are smaller than the data symbols for all points.

Surprisingly, the characteristics of these nonlinear LCST profiles obtained for NR_4_Cl salts differ from strongly hydrated metal cation salts such as CaCl_2_, MgCl_2_, and LiCl. For these metal chloride salts, the initial LCST trend is linear and a deviation in the form of an upward curvature becomes increasingly significant at higher concentrations.^33^ In contrast, these tetraalkylammonium chloride salts produce a nonlinear LCST response dominant at lower concentrations and appear to transform toward linearity at higher concentrations, with a sharp downward trend. This indicates that the salting-in mechanism valid for Ca^2+^, Mg^2+^, and Li^+^ does not hold for chloride salts of tetraalkylammonium cations. In fact, similar LCST curves have been demonstrated for sodium salts of weakly hydrated anions such as I^–^, ClO_4_^–^, or SCN^–^^16,17^ and are shown for NaSCN in Figure 2b. The underlying molecular mechanism for such salts has been shown to involve anion binding at particular susceptible sites on the macromolecule,^18,19^ and the overall LCST of the polymer fits the following model:
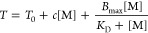
1where the LCST observed, *T*, is given by the LCST value in the absence of salt (*T*_0_) plus two terms. The first term is linear with the molar concentration of salt ([M]), with a negative coefficient *c* to express the contribution of salting-out, lowering the LCST. The second nonlinear term expresses the salting-in contribution and is derived from a Langmuir binding isotherm. Here, *B*_max_ is the maximum elevation in LCST at saturation and *K*_D_ is the apparent dissociation equilibrium constant for the binding. By applying this model to the measured LCST curves for the NR_4_Cl salts, we are able to obtain values for apparent dissociation equilibrium constants (*K*_D_) for the binding events. The apparent *K*_D_ values found are 1.2, 0.8, and 1.2 M for NBu_4_Cl, NPr_4_Cl, and NEt_4_Cl, respectively. The fit values are given in Table S1. Therefore, with increasing alkyl chain length and thus the cation size, the *K*_D_ values reach ∼1 M, indicating that the apparent binding becomes stronger with increasing cation size. The LCST experiments clearly indicate a cation-driven, saturable Langmuir-type binding of NR_4_Cl salts to PNIPAM. In order to attain a molecular-level mechanism of specific cation binding, spectroscopic ATR-FTIR and NMR measurements were performed.

The binding site for the weak metal cation-amide interactions was shown to be the amide oxygen for neutral, amide-based macromolecules.^33−35^ To determine whether the same binding site is applicable to the tetraalkylammonium cations, the amide I vibrational mode was probed in the presence and absence of 1 M salt solutions in D_2_O (heavy water) solvent. Figure 3 shows the normalized ATR-FTIR spectra of PNIPAM solutions in D_2_O and in 1 M of each of the same set of salts (NaCl, NH_4_Cl, NMe_4_Cl, NEt_4_Cl, NPr_4_Cl, NBu_4_Cl) in D_2_O media. The amide I band in D_2_O is comprised predominantly of the C=O stretch and appears at 1625 cm^–1^.^42^ In the presence of 1 M NaCl, NH_4_Cl, and NMe_4_Cl, the vibrational fingerprint remains identical with that in pure D_2_O (Figure 3a). The unaltered amide I bands indicate the absence of direct interaction. Figure 3b shows the amide I band of PNIPAM in the presence of 1 M NEt_4_Cl salt, and once again, a lack of a change in the amide I band is observed, despite the salt’s distinct nonlinear LCST influence. Figure 3c–d shows the normalized ATR-FTIR spectra in the presence of the two salts with the largest and most weakly hydrated NR_4_^+^ ions; in this case, there is a small yet distinguishable emergence of a distortion in the amide I band. This could hint at direct cation interaction with the macromolecule. However, the magnitude of the residual change (shown explicitly in Figure S2) cannot be justified with the apparent *K*_D_ values calculated from the thermodynamic LCST data; note that *K*_D_ can be interpreted as the concentration at which 50% of the binding sites are occupied in a binding event.^34^ Nevertheless, the presence of a cation in the vicinity of molecular surfaces should alter the hydration of the macromolecule. Consequently, the amide oxygen is found to not be the primary binding site of NR_4_^+^ cations on the macromolecule but may be secondarily affected in the case of the largest NR_4_^+^ cations binding at some other, nearby site. In order to further investigate the binding site for greasy cations, systematic ^1^H NMR experiments were performed.

**Figure 3:**
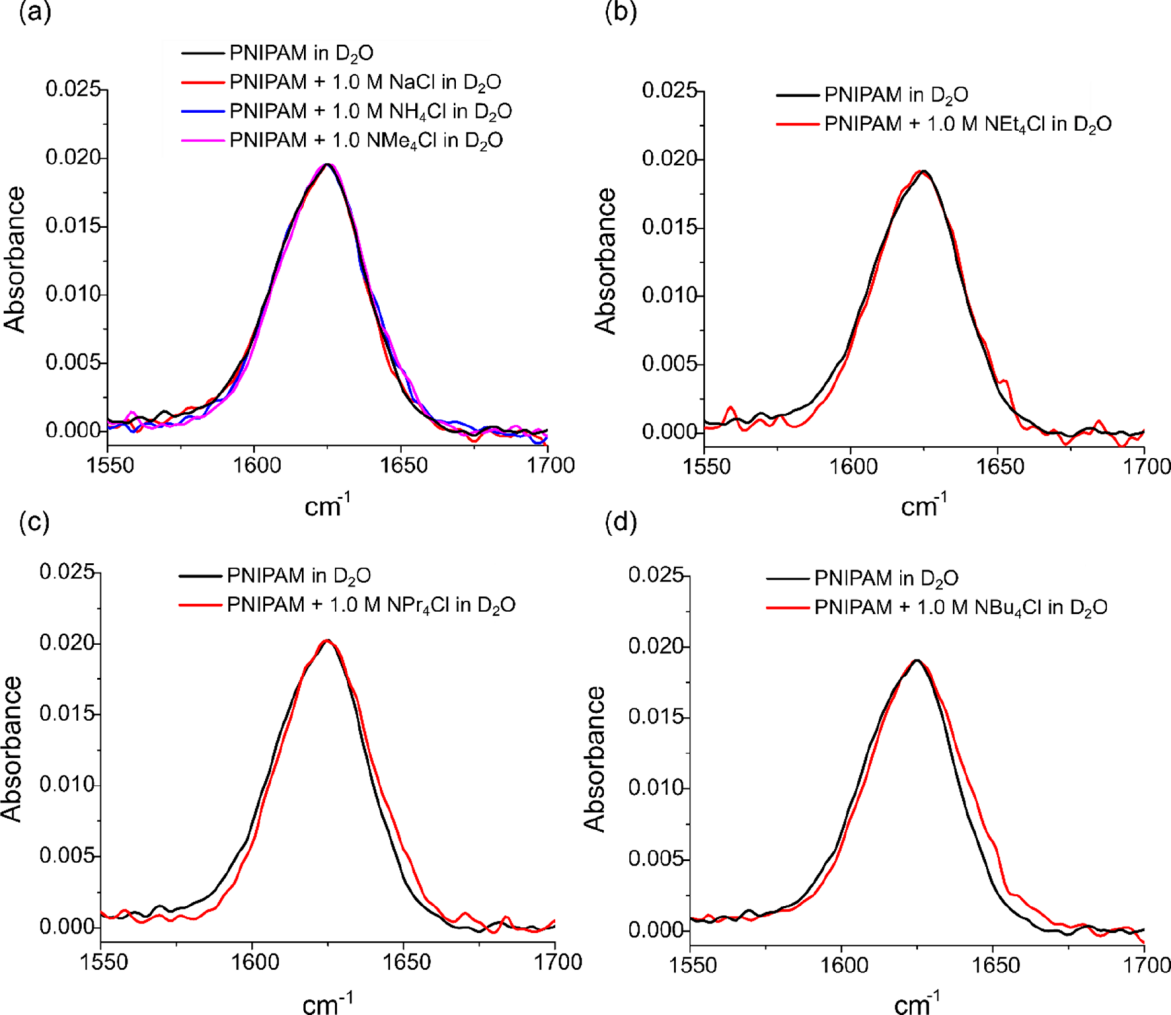
Normalized amide I ATR-FTIR bands of PNIPAM in various salt solutions in D_2_O: (a) PNIPAM in pure D_2_O as reference, PNIPAM in 1.0 M solutions of NaCl, NH_4_Cl, and NMe_4_Cl in D_2_O; (b) reference spectrum and PNIPAM in 1.0 M NEt_4_Cl solution; (c) reference spectrum and PNIPAM in 1.0 M NPr_4_Cl solution; and (d) reference spectrum and PNIPAM in 1.0 M NBu_4_Cl solution. Residuals showing the differences between bands are shown in Figure S2.

Figure 4a depicts a ^1^H NMR spectrum of PNIPAM in D_2_O (externally referenced to the DSS chemical shift reference in a coaxial insert). The macromolecule gives rise to four main resonances. Namely, the backbone methylene and −CH– (α in position to the carbonyl) groups appear at 1.58 and 2.02 ppm, respectively, and the isopropyl −CH– group (connected to the N atom), at 3.90 ppm; the isopropyl terminal −CH_3_’s appear as the large peak at 1.15 ppm, as can also be seen in the color-coded annotations in Figure 4a. When performing NMR titrations as a function of salt concentration in the range of 0 to 0.6 M, the changes in the chemical shifts (Δδ, with respect to PNIPAM in neat D_2_O) of the isopropyl terminal −CH_3_ hydrogens and the N–CH group are shown for a nonbinding (NaCl) and a binding (NBu_4_Cl) salt in Figure 4b. As can be seen, for NBu_4_Cl, the change in chemical shifts occur in a nonlinear fashion, whereas for NaCl, these sites only yield monotonic linear chemical shift trends. This is a strong indication that the chemical environment of the isopropyl group is altered as a function of salt concentration in the manner of a saturable binding site. Similar binding events have been demonstrated via numerous NMR studies^18,19,43^ and were also separately confirmed using NaSCN as a model binding salt (results are shown in comparison to NaCl, a nonbinding salt, in Figure S3). Corresponding plots for the other salts and all PNIPAM signals are included in Figure S4. The salt ions induce a net shielding effect, due to their influence on the hydrating waters and their hydrogen bonding, at all sites except for the binding site, where the saturable binding event contributes an independent partial deshielding effect, due to the electronic influence of a binding ion on the polymer.^43^ The resulting overall chemical shift curves for these sites (Figure 4b) are thus similar in shape to those for LCST measurements (Figure 2b). Accordingly, these isopropyl −CH_3_ chemical shift curves can be fit to an empirical model similar to the one utilized for ion-macromolecule binding to relate salt concentration [M] to Δδ, with analogous dissociation constant *K*_D_′ and maximum change at saturation δ_max_.
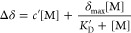
2For the isopropyl −CH_3_ groups, the *K*_D_*′* for NBu_4_Cl is 0.23 ± 0.04 M. Note that the difference between this value and the *K*_D_ obtained from LCST can be understood as the latter dissociation constant expressing an apparent, average affinity for the whole molecule, while the former pertains directly to the isopropyl binding site and is thus tighter.^44,45^ The two different *K*_D_ values for NPr_4_Cl and NBu_4_Cl are shown together in Table 1, and the full NMR fit can be found in Table S2. The curves in Figure 4c show the Langmuir isotherm residuals after the linear components are subtracted, revealing the saturable component in the terminal isopropyl −CH_3_ and N–CH chemical shift curves. Note that the backbone α- and β-sites yield solely linear chemical shift changes.

**Figure 4:**
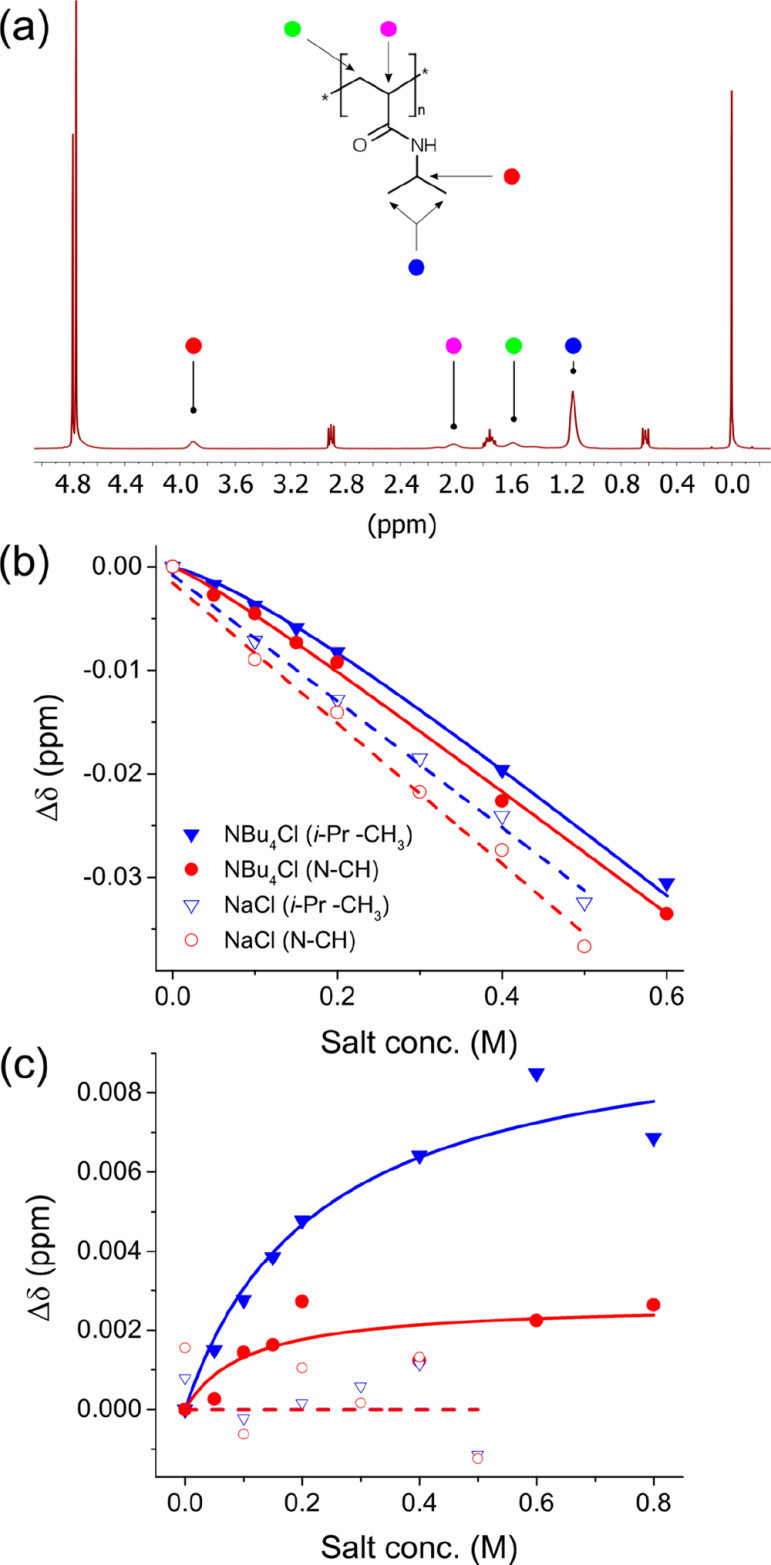
(a) ^1^H NMR spectrum of 5 mg/mL PNIPAM in D_2_O. Polymer resonances are indicated with color-coded circles on the spectrum and structure, and are described in the main text. The resonances at 0.0, 0.625, 1.755, and 2.904 ppm belong to the chemical shift reference standard DSS. The sharp peaks at 4.750 and 4.777 ppm belong to the water (HDO) in the coaxial insert and sample tubes, respectively. All ^1^H NMR measurements were conducted with external referencing, whereby the outer NMR tube (containing the sample) was fit with a smaller coaxial insert (containing the solution of DSS), such that the two solutions are measured simultaneously but do not come into contact. (b) The chemical shift curves for two positions on the polymer (the terminal −CH_3_ of the isopropyl group and the −CH site connected to the N atom) with increasing concentration of a nonbinding salt (NaCl) and a binding salt (NBu_4_Cl). The chemical shift trends are linear in the case of NaCl, indicating an effect purely driven by the influence of the salt on the solvent. The NBu_4_Cl chemical shift curves exhibit an additional nonlinear contribution, indicative of a partial deshielding effect due to the proximity of a bound ion at the respective site. The terminal −CH_3_ groups exhibit the most significant nonlinearity, whereas the N–CH group is also weakly affected. (c) Residuals for the same chemical shift series in panel (b). After fitting the data to eq 2, the linear term is subtracted to leave the nonlinear contribution, if any. Note that panels (b) and (c) share the same legend, as shown in (b).

**Table 1:** *K*_D_ Values for NPr_4_Cl and NBu_4_Cl from LCST and ^1^H-NMR Fitting^a^

salt	*K*_D_ from LCST (M)	*K*_D_′ for *i*-Pr terminal −CH_3_ from ^1^H NMR (M)
NPr_4_Cl	0.8 (0.2)	0.44 (0.05)
NBu_4_Cl	1.2 (0.15)	0.23 (0.04)

a Values in parentheses are errors.

The ^1^H NMR experiments demonstrate the binding site of NR_4_^+^ cations on the PNIPAM macromolecule: the isopropyl groups on the pendant chain. Considering the molecular size of the greasiest (NBu_4_^+^, NPr_4_^+^) cations, it can also be expected that the hydration of the remaining nearby parts of the molecule (such as the carbonyl oxygen) are also disrupted upon binding. In fact, this is apparent in the chemical shift curves for the N–CH group, which are also included in Figure 4b–c above. As can be seen, these neighboring groups also exhibit a small nonlinearity in their response, and the derived *K*_D_′ for NBu_4_Cl at this site is comparable to that of the main site, within error, which indicates that the effect should be due to the same binding event. As such, the distortion observed in the amide I bands in the presence of NBu_4_^+^ and NPr_4_^+^ cations (Figure 3c–d) should be due to some changes in the hydration of the carbonyl group and matches the blueshift that would be expected in the case of partial dehydration.^33^

In addition to the magnitude of the salting-in effect, it is curious that the salting-out efficacy of the tetraalkylammonium salts, as evident from the magnitude of the *c* parameter, also increases with an increase in cation size and hydrophobicity. As can be observed from Figure 2, with increasing alkyl chain lengths, both the nonlinear effect in the lower concentration regime and the steepness of the linear-like curve at higher concentrations is enhanced. Apparently, the greasiest tetraalkylammonium cations are both the strongest salting-in agents (at low concentrations) and salting-out agents (at higher concentrations) simultaneously. In seeking the rationale behind the salting-out ordering, a strong correlation between the entropy of hydration of the cations and the *c* parameter from eq 1 was observed (except for the smallest cations, NH_4_^+^ and Na^+^). Figure 5 plots the *c* parameter values against the molar entropy of hydration (in J·K^–1^ mol^–1^) of the cations. Surprisingly, a similar correlation was demonstrated for the salting-out efficacy of strongly hydrated anions such as SO_4_^2–^ and CO_3_^2–^.^16,17^ Such a correlation for tetraalkylammonium cations is quite unexpected; one would rather expect a correlation with the cation air/water or macromolecule/water surface tension increments. Yet, no such correlation is observed for the PNIPAM macromolecule. In fact, the tetraalkylammonium cations larger than NMe_4_^+^ actually decrease the macroscopic air–water surface tension,^42^ despite exhibiting salting-out behavior in the present system. Although the tetraalkylammonium cations are weakly hydrated in nature, they have a large number of water molecules in their hydration shells due to their larger sizes compared to monatomic cations. Thus, an overall salting-out effect is observed. Similar correlations were also observed between the *c* values and the cation polarizabilities (α, in 10^–30^ m^3^) and cationic radius (in pm) values, as shown in Figure S5. Since the phenomenological correlation goes with the ionic size and hydration entropy, the salting-out effect should be related to excluded volume-driven effects, as was also seen for large strongly hydrated molecular anions.^16,17^

**Figure 5:**
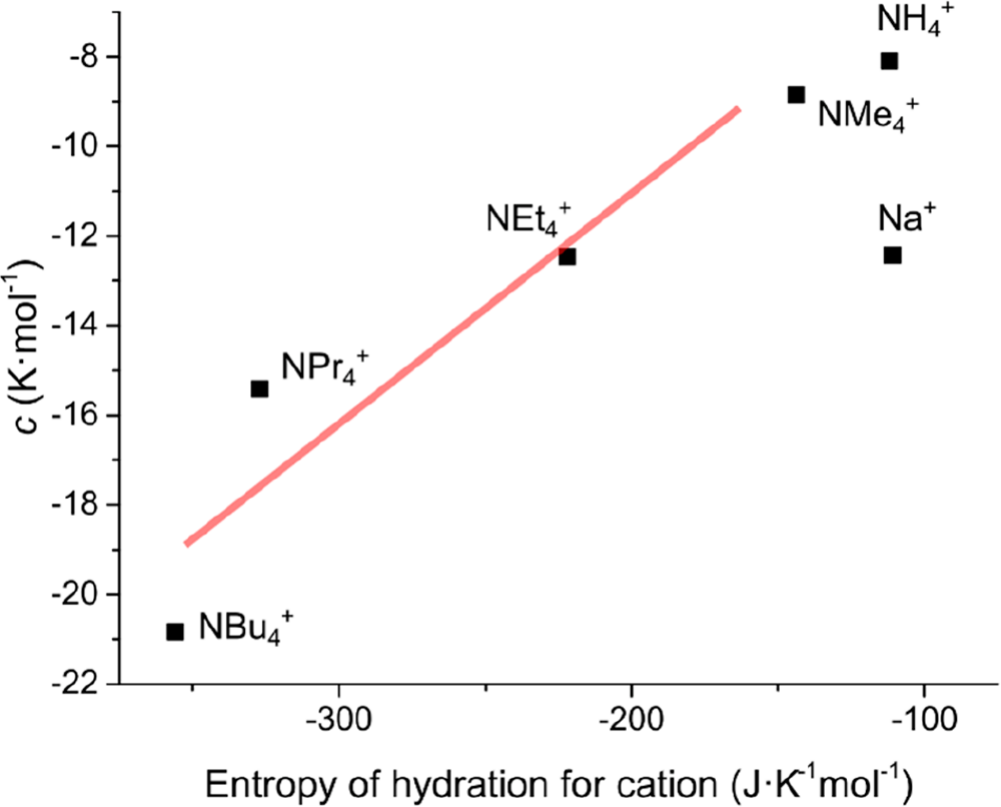
Plot of *c* values obtained by fitting the PNIPAM LCST’s for the series of salts (Figure 2b) to eq 1, against the entropy of hydration for the respective cations. Among the tetraalkylammonium cations, there is a correlation between the magnitudes of *c* values and entropies of hydration (*R*^2^ ≈ 0.89). The red line is a guide to the eye. The source for the values is ref (46).

In summary, we have herein elucidated the effects of tetraalkylammonium salts on neutral macromolecules in aqueous solution. Spectroscopic ATR-FTIR and NMR measurements have revealed that the salting-in effect of these very weakly hydrated cations is a result of direct cation binding to PNIPAM isopropyl groups. The apparent *K*_D_ values for the most weakly hydrated cations (NPr_4_^+^, NBu_4_^+^) are comparable to the apparent values demonstrated for the most weakly hydrated anions, e.g., SCN^–^, NO_3_^–^, and I^–^.^16,17^ The salting-out effect is also shown to correlate with the cation entropy of the hydration values. These results clearly demonstrate that, in contrast to the general notion that Hofmeister cations mostly form weak (statistical) interactions or are excluded from neutral macromolecule/water interfaces, the most weakly hydrated cations also bind to macromolecules. These results can inform future basic research as well as a wide variety of applications involving aqueous macromolecule solutions.

## Supporting Information Available

Experimental section, fitting results for LCST and ^1^H NMR chemical shift data, ATR-FTIR measurement residuals, ^1^H NMR chemical shift plots for all PNIPAM signals, and cation property correlation plots (PDF)
