# Supplementary material for: Greasy Cations Bind to Neutral Macromolecules in Aqueous Solution
Source: J Phys Chem Lett. 2024 Jun 5;15(23):6151–7. doi: 10.1021/acs.jpclett.4c00925 (PMC11181456; doi:10.1021/acs.jpclett.4c00925)
Supplement: Supplementary file 1 — jz4c00925_si_001.pdf [file jz4c00925_si_001.pdf]

Supporting Information

Greasy Cations Bind to Neutral Macromolecules in Aqueous Solution

Umay Eren Ertekin[1], Halil Ibrahim Okur[1,2*]

[1] Department of Chemistry, Faculty of Science, Bilkent University, 06800 Ankara, Turkey.

[2] National Nanotechnology Research Center (UNAM), Bilkent University, 06800 Ankara, Turkey

Experimental Section

Materials: All salts were purchased from Sigma-Aldrich, with purities all ≥ 99%, except for tetrapropylammonium chloride (98%) and tetrabutylammonium chloride (≥ 97%). Solutions of tetrapropylammonium chloride were filtered through cellulose acetate 0.2 μm syringe filter. The remaining salts were used as is without further purification. Poly(N-isopropylacrylamide) (PNIPAM) was purchased from Polymer Source Inc., with reported number average molecular mass of 118,500 Da and a PDI of 2.16.

Lyophilization of polymer samples: Stock solutions of the polymer were prepared and refrigerated to ensure complete dissolution. Aliquots of appropriate volume were transferred into 2 mL Eppendorf tubes/microtubes and frozen in liquid nitrogen. The samples were then dried under vacuum overnight and stored in a refrigerator until usage. To prepare a particular sample for measurement, the freeze-dried polymer samples were re-dissolved in the relevant solutions.

Measurements

LCST Measurements: Lower critical solution temperature (LCST) measurements were made at minimum in triplicate, using capillary samples in OptiMelt instrument (Stanford Research Systems). LCST points were taken as the onset of the rise in detected scattering, approximated as the intersection points of the linearized curves before and after the onset (as shown below in Figure S1, left). LCST-cosolute concentration curves were fit using OriginLab 2016 nonlinear function fitting, with Levenburg-Marquadt iteration algorithm.

ATR-FTIR Measurements: ATR-FTIR measurements were conducted using a Bruker Alpha FTIR spectrometer, equipped with a custom water-circulator/copper chamber head set-up to control the temperature of the spectrometer plate, ATR crystal and sample. The circulator head was fixed over the plate during measurement, without contact with the crystal or sample after delivery. The circulator was run in this position for at least 2 hours prior to measurement to ensure the instrument temperature was stabilized at the desired value. ATR-FTIR spectra were obtained from 32 scans with frequent background spectrum measurement.

^{1}H-NMR Measurements: ^{1}H-NMR measurements were conducted using a Bruker 400 MHz spectrometer, at ambient temperature. Samples were held in Wilmad® 5 mm thin-walled

precision 7″ NMR tubes, fitted with Wilmad® coaxial inserts for external referencing (2,2-dimethyl-2-silapentane sulfonate sodium salt (DSS) as chemical shift reference, purchased from Sigma-Aldrich, 97% purity). The reference solution was always contained in the inner insert tube, and the measurement sample in the outer tube. The two solutions were thus measured together without any physical or chemical contact. NMR spectra were obtained from 32 scans, and corrected for phase and baseline. Exact peak points of polymer bands were determined by taking the midpoint of two points on the curve at an equal height between above the half-maximum (shown below in Figure S1, right). NMR titration curves were fit to an empirical form (Equation 2 in the main text) similar to the one used for the LCST measurements. Alternatively, mathematical fitting of polymer bands to Gaussian functions produced peak positions that agreed with the midpoint method within ~0.0001 ppm (i.e., ~1% of typical differences between samples).

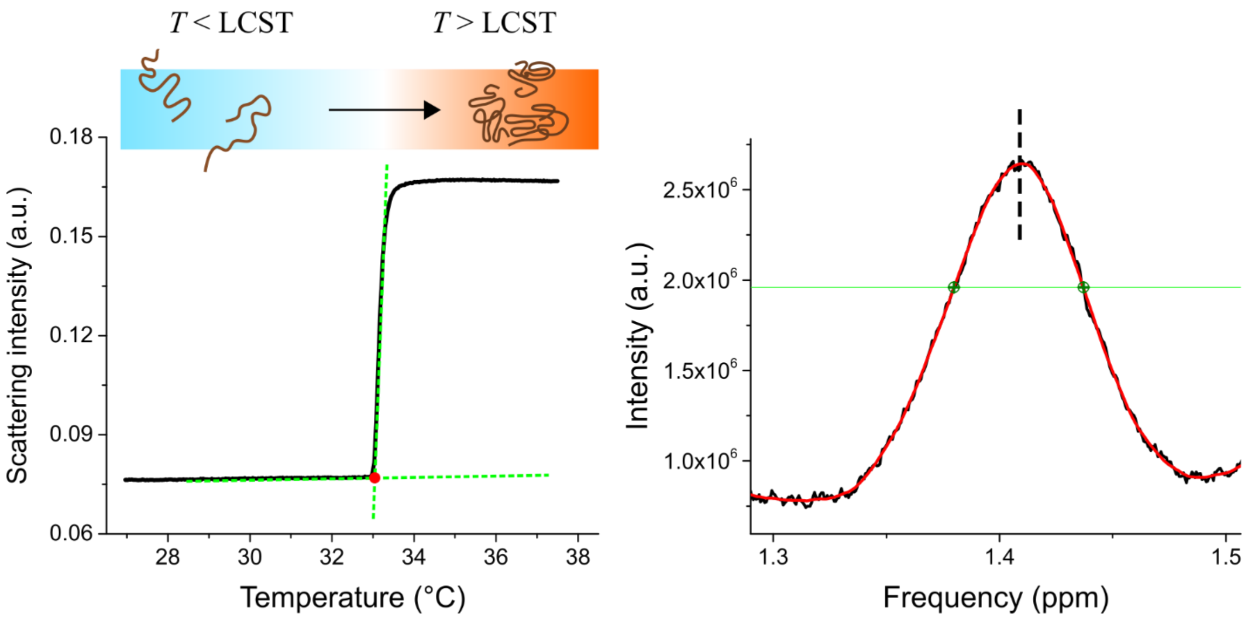

Figure S1: Left: Representative scattering intensity vs. temperature plot for PNIPAM in neat water, with tangent lines to the curve before and after the LCST onset drawn. The point of intersection (red dot) is taken as the LCST point of onset. Right: A snapshot representative of polymer band processing and peak determination. The 32-scan spectrum (black) is first smoothed (red) and then intersected with a horizontal line (green) close to the apex. The best estimate for the peak position is then taken as the average of the two intersection points between the line and the smoothed band curve.

LCST Measurements Fitting Results and Methodology

Table S1: Table of Fitted Parameters for PNIPAM LCST Curves

Salt	Concentration range	c (K·M^{-1})	K_D (M)	B_{max} (K)
NaCl	0 – 2.0 M	-12.4 (0.1)	-	-
NH_4Cl	0 – 2.0 M	-8.1 (0.1)	-	-
NMe_4Cl	0 – 1.8 M	-8.84	2.0 (0.9)	3.9 (1.1)
NEt_4Cl	0 – 1.4 M	-12.5	1.2 (0.2)	15.9 (2.1)
NPr_4Cl	0 – 1.0 M	-15.4	0.8 (0.2)	17.6 (2.4)
NBu_4Cl	0 – 1.0 M	-20.8	1.2 (0.15)	30.3 (2.7)

Table S1 above shows the fit values for the parameters c, K_D and B_{max} according to Equation 1 in the main text, for LCST values of PNIPAM in salt solutions and for the range of concentrations indicated in the second column. Values in parentheses are errors. For the first two salts, the fit is purely linear and thus there are no nonlinear terms (K_D, B_{max}).

For the tetraalkylammonium chloride salts, although the LCST measurements were carried out in the largest range of salt concentration possible, it was observed that at the highest salt concentrations tested, the LCST profiles exhibited excessive downward curvature. It is suspected that this observation is related to the known propensity of these salts to form clathrate-type solid hydrates at elevated salt concentrations.[1–4] Because this behavior is beyond the scope of our study and also incongruous with the mathematical form of Equation 1, the highest concentration data points (between one and two points) were purposefully omitted from the analysis. The range of concentrations employed for the fitting are given above in the second column of Table S1. Mathematically, it was necessary to impose a lower bound to the c parameter, which would otherwise tend to unbounded negative values in an attempt to optimize the fit. This was done by taking the tangential slope of the data between the two highest concentration points (e.g., for NBu_4Cl, the slope between 0.8 – 1.0 M), where the nonlinear term should have saturated and the slope should give the best approximation for the c parameter. A consequence of this treatment is that several of the c values shown in Table S1 have vanishingly small apparent errors; such errors are not statistically meaningful and thus were not included.

ATR-FTIR Amide I Residuals

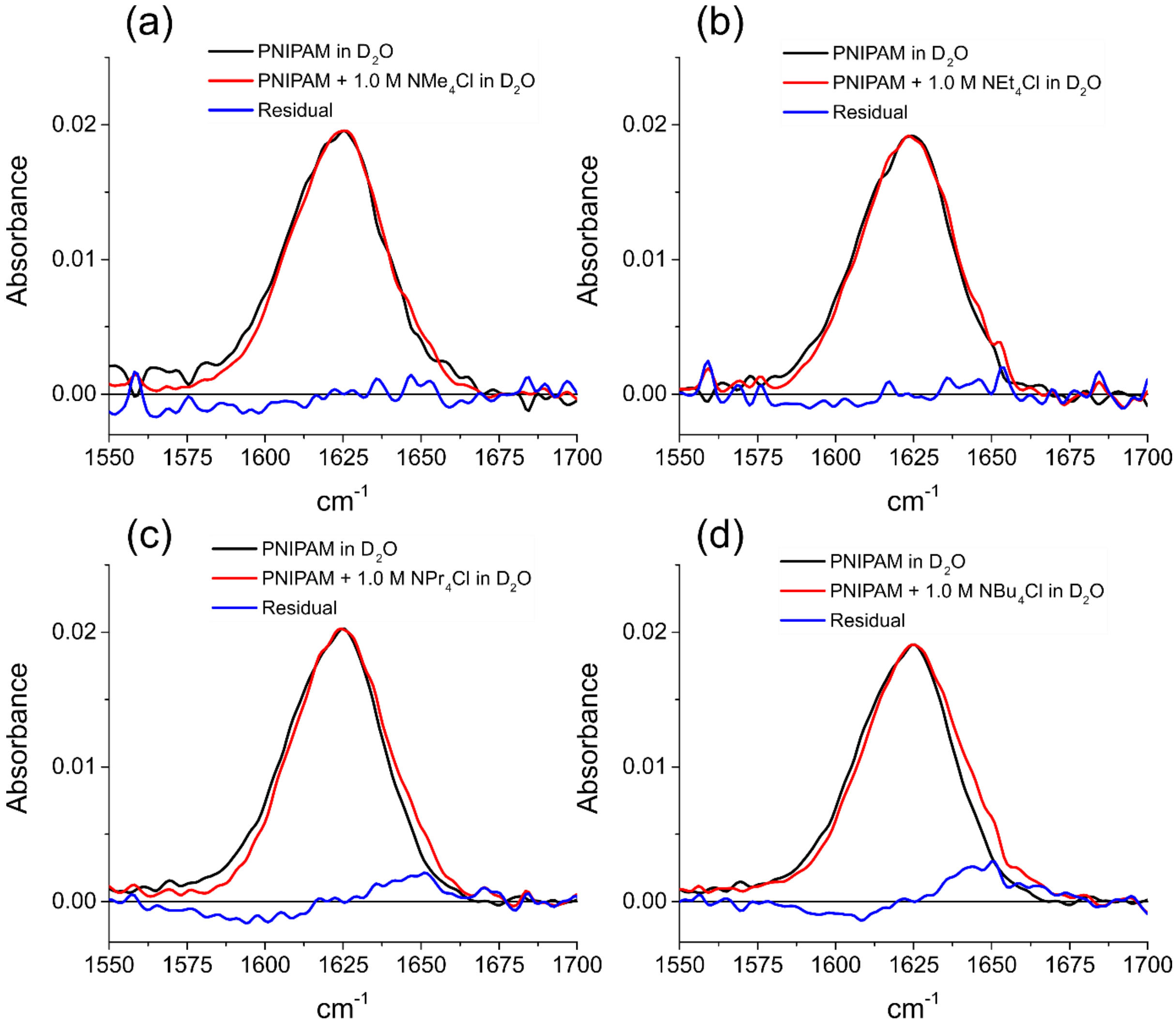

Figure S2: Normalized amide I ATR-FTIR bands of PNIPAM in various D_2O solutions: (a) PNIPAM in pure D_2O as reference, PNIPAM in 1.0 M solution of NMe_4Cl in D_2O and residual (difference between curves); (b) reference spectrum, PNIPAM in 1.0 M NEt_4Cl solution and residual; (c) reference spectrum, PNIPAM in 1.0 M NPr_4Cl solution and residual; (d) reference spectrum, PNIPAM in 1.0 M NBu_4Cl solution, and residual.

LCST and ^{1}H-NMR Chemical Shift Plots for PNIPAM – NaCl vs NaSCN

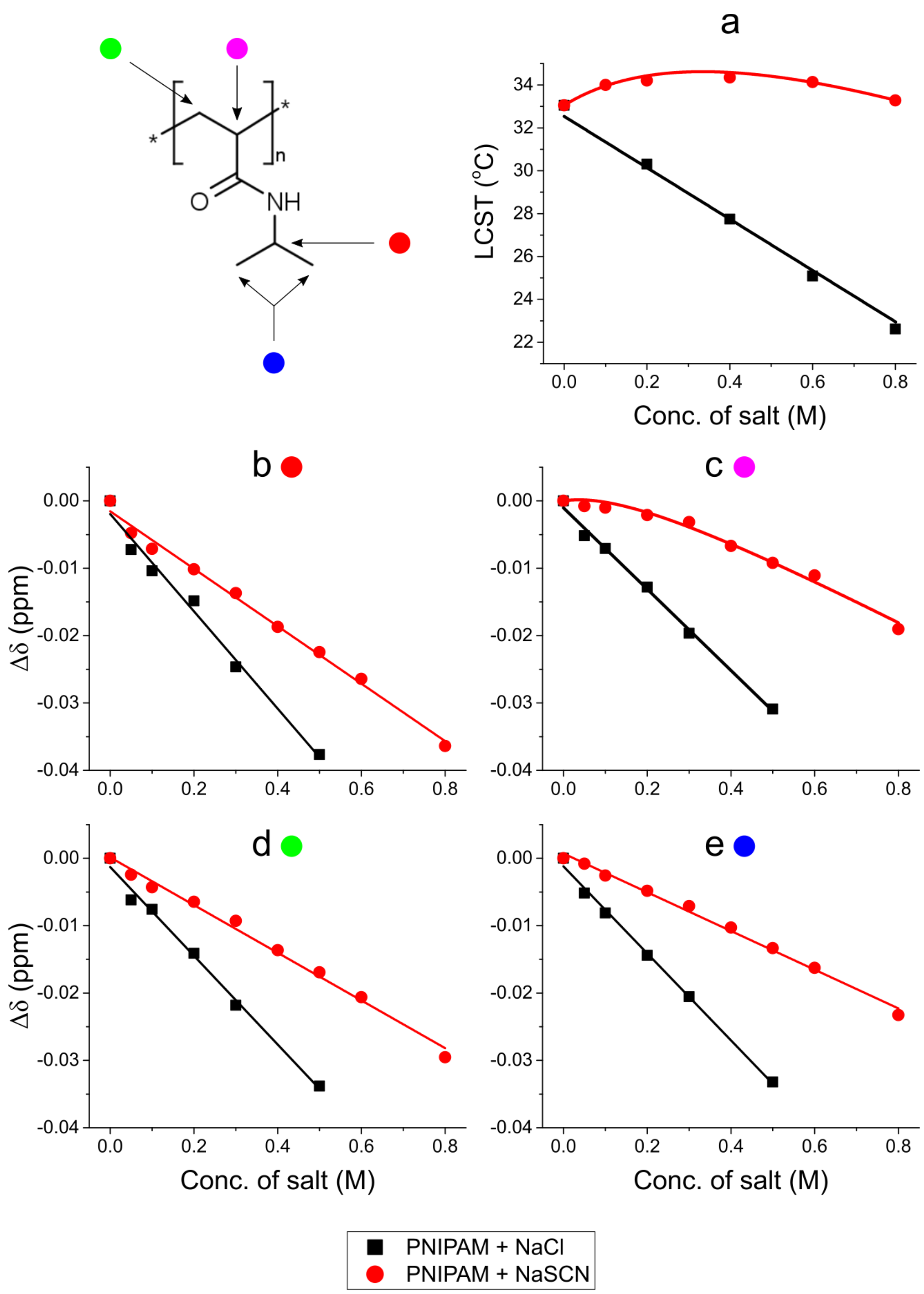

Figure S3: LCST and ^{1}H-NMR chemical shift titration curves for PNIPAM and NaCl, NaSCN salts. Structure of PNIPAM with color annotations of groups on the polymer is given at the top. (a) LCST of PNIPAM and NaCl, NaSCN salts up to 0.8 M. (b) – (e): ^{1}H-NMR chemical shift curves of the PNIPAM signals, as color-coded according to the top structure, with NaCl and NaSCN salts. The legend for (a) – (e) is shared, and shown at the bottom.

^{1}H-NMR Chemical Shift Plots for PNIPAM – Tetraalkylammonium Salts

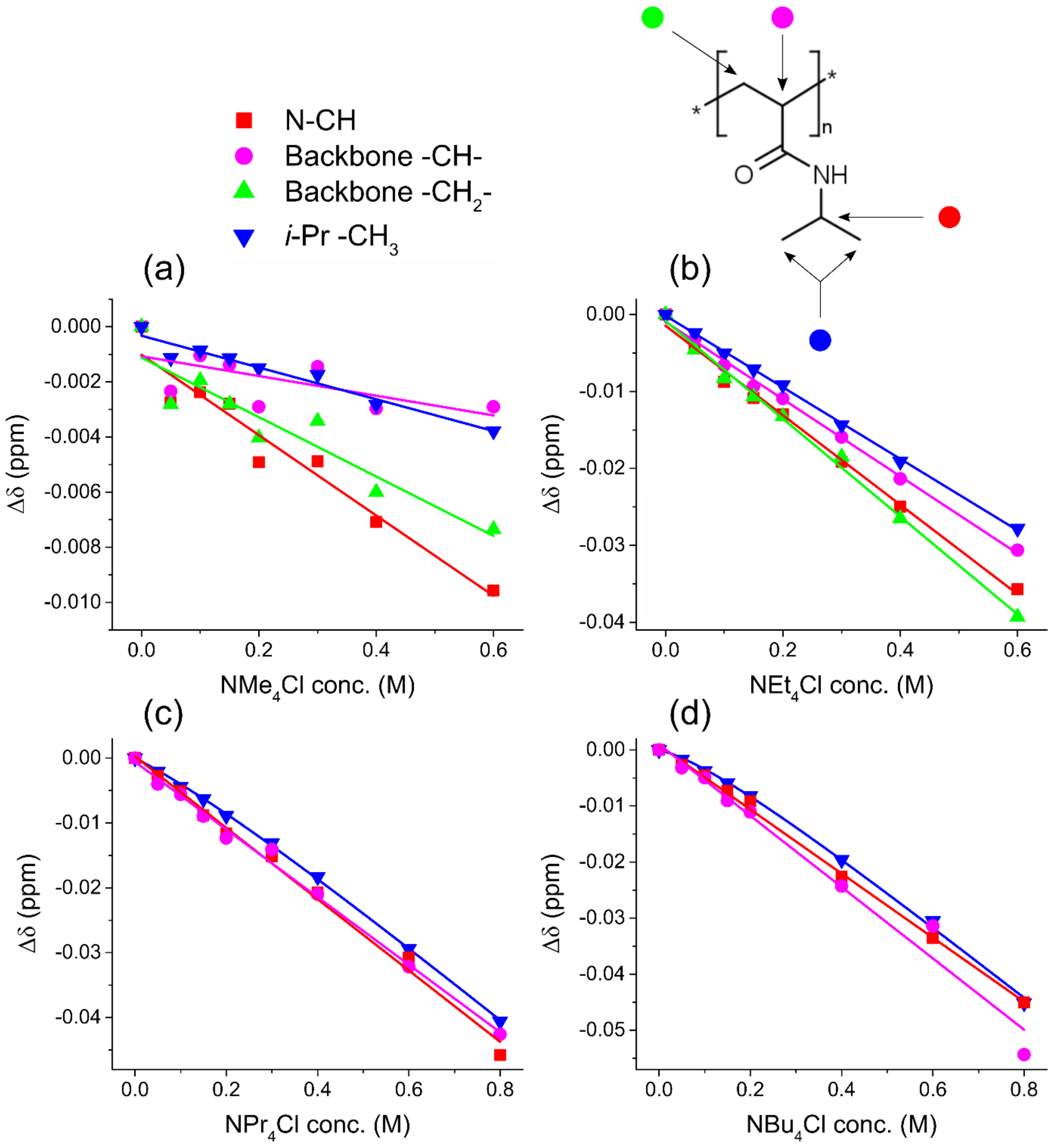

Figure S4: The ^{1}H-NMR chemical shifts for the four PNIPAM signals (legend and color-annotated structure of PNIPAM are included at the top) as a function of concentration of (a) NMe_4Cl, (b) NEt_4Cl, (c) NPr_4Cl, and (d) NBu_4Cl. Note that the backbone -CH_2- signal is absent in (c) and (d), because the signal coincides with a large salt peak and is unobservable in these cases.

Chemical Shift Measurements Fitting Results

Table S2: Table of Fitted Parameters for PNIPAM ^{1}H-NMR Chemical Shift Curves

Salt	N-CH		*i*-Pr terminal -CH_3	
	K_D' (M)	$\Delta\delta_{max}$ (ppm)	K_D' (M)	$\Delta\delta_{max}$ (ppm)
NaCl	-	-	-	-
NMe_4Cl	-	-	-	-
NEt_4Cl	-	-	-	-
NPr_4Cl	-	-	0.44 (0.05)	0.01
NBu_4Cl	0.1 (0.2)	0.003 (0.003)	0.23 (0.04)	0.01

Table S2 above tabulates the results from the fitting of N-CH and *i*-Pr terminal methyl signals' chemical shift vs. salt concentration data to Equation 2 in the main text. Values given in parentheses are errors. As can be seen, only NPr_4Cl and NBu_4Cl induce chemical shift responses that have a nonlinear component according to Equation 2 (given in the main text).

Correlation Plots: Entropy of Hydration, Polarizability, Cation Ionic Radii

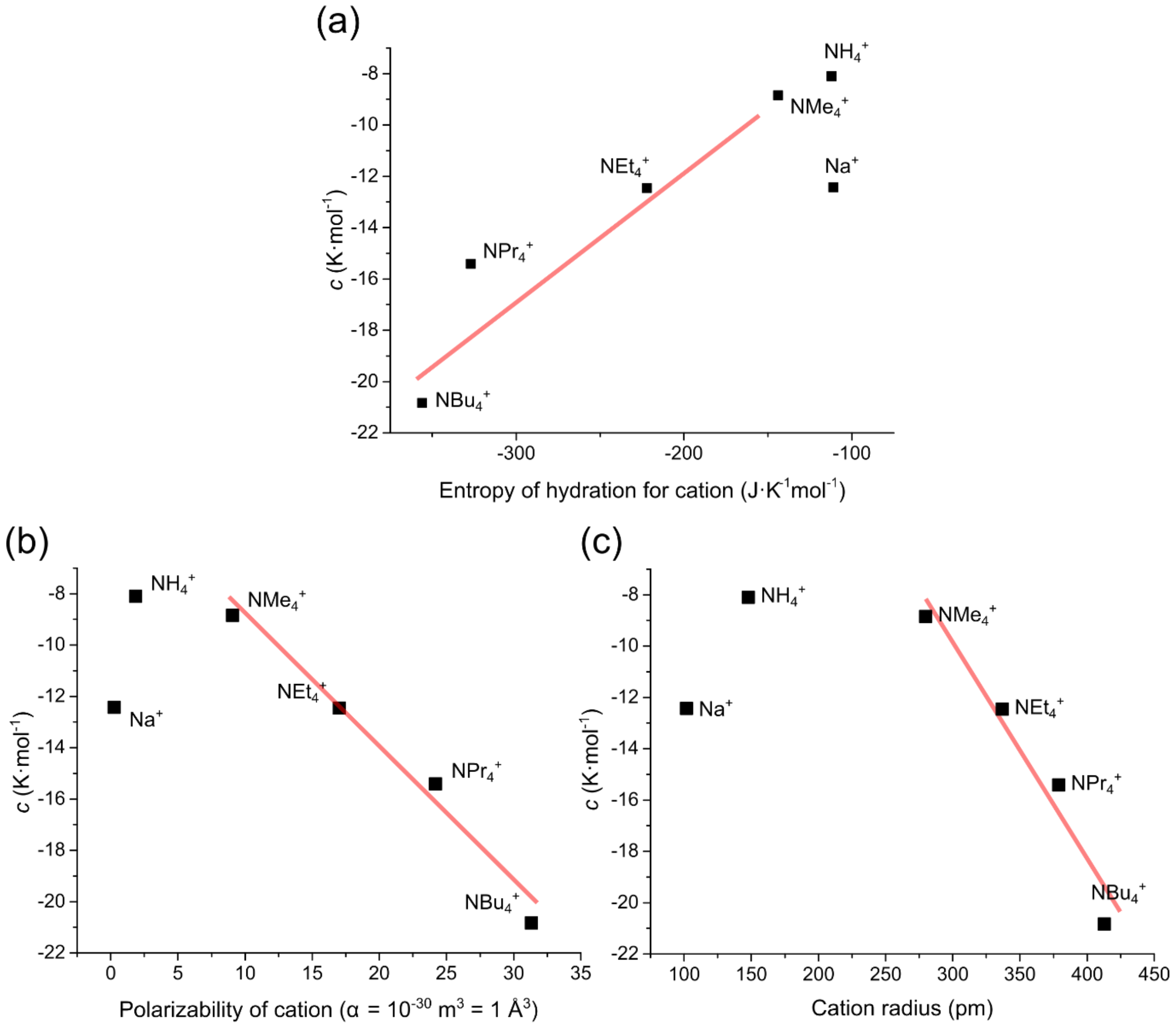

Figure S5: Plots of *c* values obtained by fitting the PNIPAM LCST's for the series of salts to Equation 2, as shown in Table S1, as correlated against: (a) the entropy of hydration ($R^2 \approx 0.89$), (b) polarizability ($R^2 \approx 0.97$), and (c) ionic radius ($R^2 \approx 0.92$), for the respective cations. The source for the cation-specific values is reference 5, which partitions experimental values between cation and anion pairs through explicit assumptions, some extra-thermodynamic. The ionic radii given for the tetraalkylammonium cations are their van der Waals radii. The red lines in each panel are meant as guides to the eye.

References:

(1) Fowler, D. L.; Loebenstein, W. V; Pall, D. B.; Kraus, C. A. Some Unusual Hydrates of Quaternary Ammonium Salts. *J. Am. Chem. Soc.* **1940**, *62* (5), 1140–1142. https://doi.org/10.1021/ja01862a039.

(2) McMullan, R.; Jeffrey, G. A. Hydrates of the Tetra *n*-butyl and Tetra *i*-amyl Quaternary Ammonium Salts. *J. Chem. Phys.* **1959**, *31* (5), 1231–1234. https://doi.org/10.1063/1.1730574.

(3) Feil, D.; Jeffrey, G. A. The Polyhedral Clathrate Hydrates, Part 2. Structure of the Hydrate of Tetra *Iso*-Amyl Ammonium Fluoride. *J. Chem. Phys.* **1961**, *35* (5), 1863–1873. https://doi.org/10.1063/1.1732158.

(4) Bonamico, M.; Jeffrey, G. A.; McMullan, R. K. Polyhedral Clathrate Hydrates. III. Structure of the Tetra *n*-Butyl Ammonium Benzoate Hydrate. *J. Chem. Phys.* **1962**, *37* (10), 2219–2231. https://doi.org/10.1063/1.1732990.

(5) Marcus, Y. *Ion Properties*; Marcel Dekker: New York, 1997.
